# Caso 3/2020 – Atresia Pulmonar, Comunicação Interventricular e Origem Anômala da Artéria Pulmonar Direita da Aorta Ascendente, em Evolução após *Shunt* Central Prévio à Esquerda, em Adulto Sintomático com 40 Anos

**DOI:** 10.36660/abc.20190487

**Published:** 2020-05-11

**Authors:** Edmar Atik, Maria Angélica Binotto, Alessandra Costa Barreto

**Affiliations:** 1 Hospital das Clínicas Faculdade de Medicina Universidade de São Paulo São PauloSP Brasil Instituto do Coração do Hospital das Clínicas da Faculdade de Medicina da Universidade de São Paulo,São Paulo, SP – Brasil

**Keywords:** Cardiopatias Congênitas, Atresia Pulmonar, Comunicação Interventricular, Artéria Pulmonar, Aorta/anormalidades, Hipertensão Pulmonar, Hipóxia, Diagnóstico por Imagem, origem da artéria pulmonar direita da aorta ascendente

## Dados clínicos

Paciente evoluiu sem sintomas desde o nascimento até a juventude, ocasião do advento de hipóxia discreta mas progressiva, que requereu a feitura de anastomose com PTFe de 8 mm entre o tronco braquiocefálico e o tronco pulmonar com 32 anos de idade. Desde então, mantém-se estável com saturação de oxigênio acima de 80 vol. O_2_%, com cansaço a moderados esforços e com palpitações precordiais. Em uso de warfarina e enalapril.

**Exame físico:** Bom estado geral, eupneico, cianose discreta de extremidades, baqueteamento digital moderado, pulsos normais nos quatro membros. Peso: 67 Kg, Alt.: 170 cm, PAMSD: 140x90 mmHg, FC: 105 bpm, saturação O_2_= 83%, Hg= 22,1 g/l, Hct= 66%.

**Precórdio:** Abaulamento torácico à esquerda, *ictus cordis* palpado para fora da linha hemiclavicular esquerda, com impulsões sistólicas nítidas na borda esternal esquerda (BEE). Bulhas cardíacas hiperfonéticas, estalido protossistólico, 2º ruído desdobrado constante, sopro sistólico discreto e suave ao longo da BEE e sopro sistólico moderado na área mitral. Fígado não palpado e pulmões limpos.

## Exames complementares

**Figura 1:**
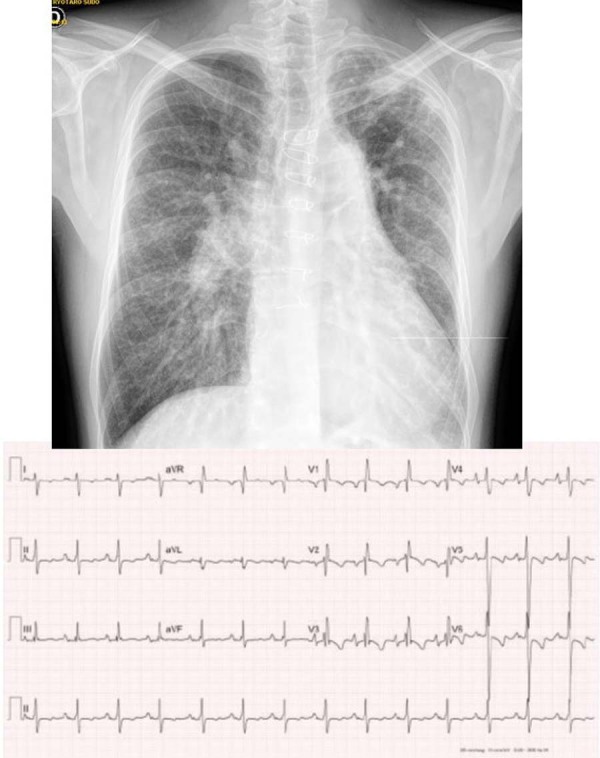
- Radiografia de tórax salienta o aumento da área cardíaca com dominância ventricular esquerda e trama vascular pulmonar aumentada no hilo direito com diminuição em direção ao lobo inferior. Ela se mostra diminuída à esquerda com vasos mais finos. Eletrocardiograma mostra a sobrecarga ventricular direita e alterações difusas da repolarização ventricular.

**E** **letrocardiograma:** Ritmo sinusal, sinais de sobrecarga ventricular direita, com com onda P apiculada a + 70° e complexo QRS com predomínio das ondas S de V4 a V6 e eixo desviado para a direita (AQRS= +110º). A onda T se mostra negativa nas derivações precordiais com alterações difusas da repolarização ventricular em todas as outras derivações ( Figura 1 ).

**Radiografia de tórax:** Aumento acentuado da área cardíaca a custa do arco ventricular esquerdo alongado, e arco médio abaulado (ICT=0,53). A trama vascular pulmonar aumentada no hilo direito se afila nos campos inferiores na expressão da hipertensão arterial pulmonar deste lado e nitidamente diminuída à esquerda com vasos finos distribuídos pelos vários lobos ( Figura 1 ).

**Ecocardiograma:** Conexão atrioventricular normal, atresia pulmonar e comunicação interventricular perimembranosa ampla de 27 mm e via de saída única com aorta (50 mm) cavalgando em mais de 50% o septo interventricular. O átrio direito é muito dilatado (volume de 67,2 ml/m^2^) assim como o átrio esquerdo (62,1 ml/m^2^). O ventrículo direito (39 mm) é dilatado e hipertrófico com disfunção discreta, com hipocinesia apical. O ventrículo esquerdo (60 mm) apresenta disfunção com fração de ejeção de 47% mas sem hipertrofia (septo=parede posterior= 10 mm). Arco aórtico à direita com a aorta abdominal à esquerda. Visualiza-se a imagem de *shunt* da anastomose entre o tronco braquiocefálico e o tronco pulmonar. A artéria pulmonar direita nasce da aorta ascendente e a esquerda é hipoplásica.

**Figura 2:**
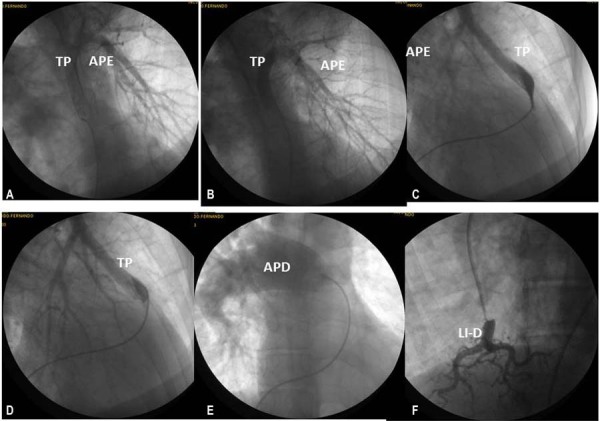
- Angiografia mostra a hipoplasia da artéria pulmonar esquerda em continuidade do tronco pulmonar hipoplásico emergindo do ventrículo direito com uma abertura anular mínima (A-D), e origem da artéria pulmonar direita dilatada e hipertensa diretamente da aorta ascendente (E,F), Artéria do lobo inferior direito mais afilada (F) . APD: artéria pulmonar direita; APE: artéria pulmonar esquerda; LI-D: lobo inferior direito.

**Cateterismo cardíaco:** Mostrou a anatomia de dupla via de saída das grandes artérias do ventrículo direito com fluxo anterógrado pulmonar exíguo (considerada como atresia pulmonar) em continuidade da artéria pulmonar esquerda hipoplásica com o tronco pulmonar também hipoplásico, artéria pulmonar direita dilatada e com hipertensão sistêmica, emergindo da aorta ascendente ( Figura 2 ).

**Diagnóstico clínico:** Atresia pulmonar, comunicação interventricular, origem anômala da artéria pulmonar direita da aorta ascendente, com hipertensão arterial pulmonar à direita e anastomose entre o tronco braquiocefálico e o tronco pulmonar, com hipoplasia pulmonar à esquerda, disfunção biventricular e sinais de hipóxia crônica em evolução, em idade adulta avançada.

**Raciocínio clínico:** Havia elementos clínicos de orientação diagnóstica da cardiopatia congênita cianogênica com diminuição do fluxo pulmonar, com mal posição arterial dada a hiperfonese de bulhas cardíacas e de atresia pulmonar em associação à comunicação interventricular. A sobrecarga de ventrículo direito no eletrocardiograma expressa a predominância deste ventrículo dada a obstrução pulmonar acentuada. O diagnóstico da origem anômala da artéria pulmonar direita da aorta ascendente ocasionando hipertensão arterial pulmonar ipsilateral poderia ter sido cogitado pela valorização e análise adequada da trama vascular pulmonar, acentuadamente dilatada. O grau discreto da hipóxia com saturação de oxigênio acima de 80% guarda relação com essa trama vascular pulmonar aumentada na radiografia de tórax, apesar da doença vascular pulmonar. Mas, mesmo assim em adultos, proporciona um aumento considerável das hemácias e da sua concentração em relação à do soro. O diagnóstico da anomalia foi bem estabelecido pela ecocardiografia, e principalmente pela angiografia.

**Diagnóstico diferencial:** Outras cardiopatias que se acompanham de comunicação interventricular e de atresia pulmonar apresentam outros elementos que as diferenciam nos exames complementares usuais, como na dupla via de entrada de ventrículo esquerdo ou direito, nas atresias das valvas atrioventriculares, na transposição corrigida das grandes artérias e em outras mais raras. O contraste das duas circulações pulmonares, mais acentuada à direita e diminuída à esquerda poderia orientar à presença de estenose deste lado e hiperfluxo por circulação colateral no outro lado. Mas nessa condição haveria sopro contínuo nítido principalmente no dorso à direita. Daí a origem da artéria pulmonar direita da aorta ascendente poderia ser cogitada clinicamente, mesmo antes do diagnóstico anatômico firmado.

**Conduta:** Apesar do balanceamento dos fluxos pulmonar e sistêmico ao longo do tempo, e com sinais de hipoxemia e de disfunção miocárdica se pressente a necessidade de aumentar um pouco mais o fluxo pulmonar para melhorar a qualidade de vida com melhor tolerância física. Em face da complexidade anatômica, da hipertensão arterial pulmonar à direita e da disfunção biventricular presentes, houve a consideração da conduta expectante clínica, apesar de riscos evolutivos inerentes.

**Comentários:** A evolução natural deste paciente até a idade adulta nos salienta elementos desfavoráveis, relacionados à doença vascular pulmonar à direita, dada a anomalia da artéria pulmonar direita com origem direta da aorta ascendente, com nítida transmissão da pressão arterial sistêmica. Ademais, a hipóxia crônica ter ocasionado a disfunção biventricular, além das outras lesões responsáveis pela maior hipertrofia ventricular como a atresia pulmonar e a própria dextroposição aórtica. Houve melhora clínica após a anastomose sistêmico-pulmonar, o que foi de boa conduta na atenuação da hipóxia. No entanto, em face de outros parâmetros, espera-se a deterioração mais rápida com aparecimento de complicações de trombose, embolia, arritmias, insuficiência cardíaca e até eventos súbitos. Por outro lado, a conduta expectante considerada foi a mais plausível em vista do risco cirúrgico elevado e considerável nesta faixa etária, além da hipertensão arterial pulmonar presente à direita, e sem solução funcional adequada.^1^

Pergunta-se, em casos semelhantes na idade infantil, se não seria mais conveniente a tentativa da correção mais precoce. Sem dúvida ela sempre deve ser considerada em estados distintos a moldar uma anatomia adequada e favorável à dinâmica sanguínea.^2^

Esta associação de defeitos é extremamente rara haja vista que na literatura são descritos três casos semelhantes e todos da origem anômala da artéria pulmonar esquerda da aorta ascendente.^1 - 3^
