# Supplementary material for: Caso 3/2020 – Atresia Pulmonar, Comunicação Interventricular e Origem Anômala da Artéria Pulmonar Direita da Aorta Ascendente, em Evolução após Shunt Central Prévio à Esquerda, em Adulto Sintomático com 40 Anos
Source: Arq Bras Cardiol. 2020 May 11;114(4 Suppl 1):43–6. [Article in Portuguese] doi: 10.36660/abc.20190487 (PMC8149107; doi:10.36660/abc.20190487)
Supplement: Translation

# Translation: Case 3/2020 – Pulmonary Atresia, Interventricular Communication and Anomalous Origin of the Right Pulmonary Artery from the Ascending Aorta developing after Prior Left Central Shunt, in a Symptomatic 40-year-old Adult.

**Authors:** Edmar Atik, Maria Angélica Binotto, Alessandra Costa Barreto

**Affiliations:** 1 Brazil Instituto do Coração do Hospital das Clínicas da Faculdade de Medicina da Universidade de São Paulo, São Paulo, SP – Brazil

**Keywords:** Heart Defects, Congenital, Pulmonary Atresia, Heart Septal Defects, Ventricular, Pulmonary Artery, Aorta/abnormalities, Hypertension, Pulmonary, Hypoxia, Diagnostic, Imaging, origin of right pulmonary artery from ascending aorta

**Clinical Data:** The patient had no symptoms from birth to young adulthood, when he developed more progressive mild hypoxia, which required an anastomosis with 8 mm PTFE graft between the brachiocephalic trunk and the pulmonary trunk, at the age of 32. Since then, the patient remains stable with oxygen saturation levels above 80%, fatigue on moderate exertion and precordial palpitations. He used warfarin and enalapril.

**Physical examination:** Good overall health status, eupneic, mild cyanosis in the extremities, moderate digital clubbing, normal pulses in the 4 limbs. Weight: 67 Kg; height: 170 cm; right upper limb blood pressure: 140x90 mmHg; HR: 105 bpm; O2 Saturation = 83%, Hg= 22.1 g/l; Hct= 66%.

**Precordium:** Left chest bulge, apex beat palpable outside the left hemiclavicular line, with clear systolic impulses at the left sternal border (LSB). Hyperphonetic heart sounds, protosystolic click, constant split 2nd heart sound, discrete and mild systolic murmur along the LSB and moderate continuous murmur in the mitral region. The liver was not palpable, and the lungs were clear.

## Complementary examinations

**Electrocardiogram:** Sinus rhythm, signs of right cavity overload, with apiculate P wave + 70º, QRS complex showing predominance of S waves from V4 to V6 and axis deviated to the right (AQRS= +110°). The T wave was negative in the precordial leads with diffuse ventricular repolarization changes in all other leads ( Figure 1 ).

**Figure 1:** - Chest X-ray showing enlarged cardiac area with left ventricular dominance and increased pulmonary vascular network in the right hila, with a reduction towards the lower lobe. It is reduced to the left with thinner blood vessels. Electrocardiogram shows right cavity overload and diffuse ventricular repolarization changes.

**Chest X-ray:** Marked enlargement of the cardiac area on account of the elongated left ventricular arch, and bulging middle arch (CTI=0.53). The increased pulmonary vascular network in the right hila tapers off towards the lower lobes, in pulmonary artery hypertension expression, and is clearly reduced on the left side with thin blood vessels coursing through several lobes ( Figure 1 ).

**Echocardiogram:** Normal atrioventricular connection, pulmonary atresia, wide perimembranous interventricular communication (27 mm) and single outflow tract with aorta (50 mm), overriding the interventricular septum for more than 50%. The right atrium is very dilated (RAV=67.2 ml/m^2^), as well as the left atrium (62.1 ml/m^2^). The right ventricle (39 mm) is dilated and hypertrophic, with moderate dysfunction and apical hypokinesia. The left ventricle (60 mm) shows dysfunction with ejection fraction of 47%, but without hypertrophy (septum=posterior wall= 10 mm). Right-sided aortic arch with left-sided abdominal aorta. Shunt anastomosis between the brachiocephalic trunk and the pulmonary trunk is visualized. The right pulmonary artery originates from the ascending aorta, and the left one is hypoplastic.

**Cardiac catheterization:** It revealed the anatomy of a double outlet of the great arteries from the right ventricle with minimal antegrade pulmonary blood flow (considered as pulmonary atresia), hypoplastic left pulmonary artery in continuity, associated with hypoplasia of the pulmonary trunk, dilated right pulmonary artery and systemic hypertension originating from the ascending aorta ( Figure 2 ).

**Figure 2:** - Angiotomography showing hypoplasia of the left pulmonary artery, with hypoplastic pulmonary artery continuity emerging from the right ventricle with minimum annular opening (A-D), and origin from dilated and hypertensive right pulmonary artery directly from the ascending aorta (E,F), Thinner inferior right lobe artery (F). RPA: right pulmonary artery; LPA: left pulmonary artery; IL-R: inferior right lobe.

**Clinical diagnosis:** Pulmonary atresia, interventricular communication, anomalous origin of the right pulmonary artery from the ascending aorta, right-sided pulmonary artery hypertension and anastomosis between the brachiocephalic trunk and the pulmonary trunk, with left pulmonary hypoplasia, biventricular dysfunction and signs of chronic progressive hypoxia in later adulthood.

**Clinical reasoning:** There were clinical elements leading to a diagnosis of cyanogenic congenital heart disease with decreased pulmonary flow, with arterial malposition considering the hyperphonetic heart sounds and pulmonary atresia in association with interventricular communication. The right ventricular overload on the electrocardiogram demonstrates the predominance of this ventricle, given the marked pulmonary obstruction. The diagnosis of anomalous origin of the right pulmonary artery from the ascending aorta, leading to ipsilateral pulmonary artery hypertension, could be considered by means of the appreciation and adequate analysis of the markedly dilated pulmonary vascular network. The discrete degree of hypoxia with oxygen saturation of approximately 80% is associated with this increased pulmonary vascular network on the chest X-ray, despite the pulmonary vascular disease. Still, even in adults, it provides a considerable increase in red blood cells and their levels in relation to that of serum. The diagnosis of the anomaly was well established by the echocardiography and mainly by the angiography.

**Differential diagnosis:** Other heart diseases that accompany interventricular communication and pulmonary atresia show other features that differentiate them in the usual complementary exams, such as the double inlet left or right ventricle, in atrioventricular valve atresia, corrected transposition of the great arteries and in rarer diseases. The contrasts in the two pulmonary circulations, more marked on the right and reduced on the left, could orient towards the presence of stenosis on this side and hyperflow due to collateral circulation on the other side. However, in this condition there would be a clear continuous murmur, mainly at the right back. Thus, origin of the right pulmonary artery from the ascending aorta could be clinically considered, even before the anatomical diagnosis.

**Conduct:** Despite the balance of the pulmonary and systemic flows over time, with signs of hypoxemia and myocardial dysfunction, the need to increase pulmonary flow to improve quality of life with better physical tolerance is presumed. In face of the anatomical complexity, right-sided pulmonary artery hypertension and biventricular dysfunction, the consideration of the expectant conduct was not ruled out, despite the development risks involved.

**Comments:** The natural evolution of this patient until the adult age highlights unfavorable elements, related to right-sided pulmonary vascular disease, given the anomalous origin of the right pulmonary artery from the ascending aorta, with clear transmission of systemic blood pressure. Furthermore, chronic hypoxia induced biventricular dysfunction, in addition to other injuries responsible for the greatest ventricular hypertrophy, such as pulmonary atresia and even aortic dextroposition. There was clinical improvement after the performance of systemic-pulmonary anastomosis, which was a useful strategy for hypoxia mitigation. However, in face of the other parameters, a quicker deterioration is expected, with the emergence of thrombosis, embolism, arrhythmias, heart failure complications and even sudden events. On the other hand, the expectant conduct considered was the most plausible in view of the high and considerable surgical risk in this age group, in addition to right-sided pulmonary arterial hypertension, and no adequate functional solution.^1^

The question is, in similar cases in childhood, whether it would be more convenient to attempt an earlier correction. Undoubtedly, it should always be considered at different conditions to create an anatomic shape that is adequate and favorable to blood dynamics.^2^

The combination of these defects is extremely rare since in the literature three similar cases are described, all of them involving the anomalous origin of the left pulmonary artery from the ascending aorta.^1 - 3^
